# Team dynamics and clinician’s experience influence decision-making during Upper-GI multidisciplinary team meetings: A multiple case study

**DOI:** 10.3389/fonc.2022.1003506

**Published:** 2022-10-18

**Authors:** J.C.H.B.M. Luijten, M.J. Westerman, G.A.P. Nieuwenhuijzen, J.E.W. Walraven, M.N. Sosef, L.V. Beerepoot, R. van Hillegersberg, K. Muller, R. Hoekstra, J.J.G.H.M. Bergman, P.D. Siersema, H.W.M. van Laarhoven, C. Rosman, L. Brom, P.A.J. Vissers, R.H.A. Verhoeven

**Affiliations:** ^1^ Department of Research & Development, Netherlands Comprehensive Cancer Organization (IKNL), Utrecht, Netherlands; ^2^ Department of Epidemiology and Data Science Amsterdam University Medical Center (UMC), Amsterdam, Netherlands; ^3^ Department of Surgery, Catharina Hospital, Eindhoven, Netherlands; ^4^ Department of Medical Oncology, Radboudumc, Nijmegen, Netherlands; ^5^ Department of Surgery, Zuyderland Hospital, Heerlen, Netherlands; ^6^ Department of Medical Oncology, Elisabeth Tweesteden Hospital, Tilburg, Netherlands; ^7^ Department of Surgery, Utrecht University Medical Center (UMC), Utrecht, Netherlands; ^8^ Department of Radiation Oncology, Radiotherapy Group, Deventer, Netherlands; ^9^ Department of Medical Oncology, Hospital group Twente (ZGT), Almelo, Netherlands; ^10^ Department of Gastroenterology, Cancer Center Amsterdam, Amsterdam University Medical Center (UMC), University of Amsterdam, Amsterdam, Netherlands; ^11^ Department of Gastroenterology and Hepatology, Radboudumc, Nijmegen, Netherlands; ^12^ Department of Medical Oncology, Cancer Center Amsterdam, Amsterdam University Medical Center (UMC), University of Amsterdam, Amsterdam, Netherlands; ^13^ Department of Surgery, Radboudumc, Nijmegen, Netherlands

**Keywords:** multidisciplinary team meeting (MDT), team dynamics, upper-GI cancer, experience, clinicians

## Abstract

**Background:**

The probability of undergoing treatment with curative intent for esophagogastric cancer has been shown to vary considerately between hospitals of diagnosis. Little is known about the factors that attribute to this variation. Since clinical decision making (CDM) partially takes place during an MDTM, the aim of this qualitative study was to assess clinician’s perspectives regarding facilitators and barriers associated with CDM during MDTM, and second, to identify factors associated with CDM during an MDTM that may potentially explain differences in hospital practice.

**Methods:**

A multiple case study design was conducted. The thematic content analysis of this qualitative study, focused on 16 MDTM observations, 30 semi-structured interviews with clinicians and seven focus groups with clinicians to complement the collected data. Interviews were transcribed *ad verbatim* and coded.

**Results:**

Factors regarding team dynamics that were raised as aspects attributing to CDM were clinician’s personal characteristics such as ambition and the intention to be innovative. Clinician’s convictions regarding a certain treatment and its outcomes and previous experiences with treatment outcomes, and team dynamics within the MDTM influenced CDM. In addition, a continuum was illustrated. At one end of the continuum, teams tended to be more conservative, following the guidelines more strictly, versus the opposite in which hospitals tended towards a more invasive approach maximizing the probability of curation.

**Conclusion:**

This study contributes to the awareness that variation in team dynamics influences CDM during an MDTM.

## Introduction

Patients with esophagogastric cancer have a poor prognosis and only half of diagnosed patients are amenable to curative treatment (1–4). The probability of undergoing treatment with curative intent for esophagogastric cancer by the hospital of diagnosis varies significantly in the Netherlands (5–7). Moreover, a higher probability of undergoing treatment with curative intent, stratified according to the hospital of diagnosis, is associated with better survival (5–7). Patient-and tumor-related factors could only partially explain variability in treatment practice (5–7). Hence, variability is likely due to multiple factors at different levels, such as clinical decision-making (CDM) during the outpatient clinic visit, the organization of clinical pathways, and CDM during multi-disciplinary team meetings (MDTM) (8–11). However, it is currently not clear which factors contribute to CDM during an MDTM.

As a result of the centralization of esophagogastric cancer surgery in the Netherlands, most resection centers have implemented regional networks and expert upper-gastrointestinal (GI) MDTMs. Expert MDTMs facilitate consensual decision-making, regional uniformity of proposed care, and uniform adherence to clinical practice guidelines, and team collaboration (12, 13). Although CDM is the essence of everyday clinical practice and is a pivotal part of oncological care, there is limited information regarding the factors that contribute to CDM during an MDTM. Previous studies have reported that CDM may be influenced by a physician’s individual experience and personality (14–16). In addition, multi-disciplinary CDM has been reported to be influenced by the behavior and performance of the individual team members (17–19). Nonetheless, previous studies only partially report factors potentially explaining variability in hospital practice. Hence, a greater understanding of facilitators and barriers to CDM during an upper-GI cancer MDTM may complement and clarify factors explaining the observed variability in clinical practice. The aim of this qualitative study was to identify factors associated with CDM that may potentially explain differences in hospital practice.

## Materials and methods

### Study design

This study was part of a mixed methods multiple case study investigating the underlying causes of variabilities in hospital practice in the curative treatment of esophagogastric cancer, known as the VARIATE project (outlined in **Textbox 1**). The present study focused on identifying facilitators and barriers influencing CDM during MDTM, as well as factors potentially explaining variability in hospital practice. This study was funded by the Dutch Cancer Society (Project No. 10895).

Textbox 1 **The VARIATE study: A mixed methods multiple case study combining qualitative and quantitative research**

All patients diagnosed with esophageal and gastric cancer in the Netherlands are registered in the Netherlands Cancer Registry (NCR). Previous multivariable multilevel analyses of patients diagnosed during the period 2015–2017 have shown that the probability of receiving treatment with curative intent differed according to the hospital of diagnosis (5). Hospitals were divided into three tertiles: low, middle, or high probability of undergoing treatment with curative intent. Patients diagnosed in a hospital with a high probability of receiving treatment with curative intent had a significant better long-term survival (5). In order to obtain in-depth information and knowledge of the underlying mechanisms of hospital practice variation in proposing treatment with curative intent the VARIATE project (VariAtion in the cuRatIve treatment of esophAgeal and gasTric cancEr) was developed, which was financed by the Dutch Cancer Society.

Receiving treatment with curative intent was defined as endoscopic or surgical resection, initiation of surgery (without resection), or definitive chemoradiation (external beam radiotherapy and concurrent chemotherapy; including initiation of chemoradiation). Palliative treatment was defined as: palliative systemic therapy, palliative radiotherapy, and best supportive care.

**Design:**

The VARIATE project is a mixed methods multiple case study, which combines qualitative and quantitative research. A selective sample (20) of eight cases (i.e., hospitals) participated. These hospitals were a representative sample of Dutch hospitals regarding the probability of offering treatment with curative intent (low (L), low or middle for gastric or esophageal cancer (L/M), and high (H)), hospital type, size, and geographical location.

*Recruitment:* Surgeons or medical oncologists from 11 different hospitals were invited by email. After interest was voiced, JL presented the study during the MDTM of the eight interested hospitals to assess the interest of the multidisciplinary team. All hospitals and team members who saw the presentation wished to participate in this study.

Our study used an iterative approach for qualitative data collection and analyses, data collection consisted of:

1. Observations of (Upper-GI specific) MDTMs (2–4 MDTMs per hospital) and outpatient clinic visits (minimum of 2 outpatient clinic visits per hospital)
2. Semi-structured interviews (n=30) with clinicians involved in the multidisciplinary care for esophageal and gastric cancer *(i.e., surgeons ( n=8), medical oncologists (n=6), radiation oncologists (n=5), gastroenterologists (n=6), and case managers (n=5))*
3. Focus groups with clinicians in order to validate and further enrich the results of their own hospital (n=7).
4. Focus groups with patients diagnosed with potentially curable esophageal- or gastric cancer were organized to explore factors related to their treatment choices (n=3: low, middle and high probability hospital).

Based on the analysis of the first 3 hospitals the following decisions regarding the quantitative and qualitative data collection in the further hospitals were made:

1. Depending on the emerging topics from previous interviews the topic list was modified *(more focus on: MDTMs, cases of doubt, shared decision making)*.
2. Clinicians in the other five hospitals were selected for interviewing through emergent sampling *(i.e., gastroenterologist that did not treat early carcinomas were not invited for participation, recent new members in multidisciplinary teams were not invited for participation).*
3. In a subsample of esophagogastric cancer patients diagnosed from 2015–2017 additional quantitative data was gathered, for esophageal cancer additional data was gathered in 38 hospitals and for gastric cancer 66 hospitals *(i.e., data was gathered by the NCR regarding diagnostics, the MDTM treatment proposal and outpatient clinic visits)* in order to gain insight in clinical pathways and alterations in MDTM treatment proposal.

The VARIATE-project focusses on the organization of clinical pathways as well as MDTMs, and the outpatient clinic visit.

**Analyses:**

1. Qualitative analyses: Interviews were audio recorded, transcribed per verbatim and summarized (all by JL) and shared with the interviewed clinicians to serve as a member check. Next, the interviews were reviewed and coded, using open coding as described by Strauss and Corbin (21). To minimize subjectivity the first 11 transcripts were independently coded by two researchers (JL, PV) and discussed until a consensus was reached (22). The remaining 19 transcripts were coded by JL. A summary was written for each interview and each hospital. Using thematic content analyses emerging themes were found (23). Simultaneously, through a constant comparison across and within cases, relations were searched for and themes were identified (24). The core study group (JL, PV, RV, GN) met weekly to discuss analyses, refine the codebook and identify emerging themes. The coding process was facilitated by Atlas.ti, version 8.0 (ATLAS.ti Scientific Software Development GmbH, Berlin, Germany).
2. Quantitative analyses: Quantitative data was analyzed according to the probability of receiving treatment with curative intent using SAS® version 9.4 (SAS Institute, Cary, North Carolina, USA). A p-value below 0.05 was considered statistically significant.

### Setting and procedures

Eight hospitals (cases) were selected to participate in the study (see methods in the **Textbox 1**) based on their likelihood of offering treatment with curative intent (low [L], n=2, low/middle [L/M], n=2 and high probability [H], n=4) (5), hospital type (academic resection hospitals [n=3], non-academic resection hospitals [n=4], and referring hospital [n=1]), geographical location, and hospital size in the Netherlands (deviant case sampling) (25). A detailed description regarding the probability of offering treatment with curative intent classification was described in a previous study (5).

From January 2019 to November 2020, MDTM observations and interviews with clinicians and focus groups (FGs) were conducted. Sampling and data collection evolved during the course of the study (**Textbox 1**). All data were collected by a medical doctor (JL), who was trained to interview and organize each FG, and who analyzed the data together with two experienced researchers in the field of qualitative research (LB, MW). At the first three hospitals, upper-GI cancer care involved a team of medical oncologists, surgeons, radiation oncologists, gastroenterologists, and case managers, who were observed during the outpatient clinical visits and interviewed. Clinicians at the other five hospitals were selected for interview by emergent sampling, which implies that sampling decisions were made during the process of data collection as the study progressed (**Textbox 1**) (26).

### Data collection

#### Observations

In total, 16 MDTMs were observed at seven resection hospitals. At one referral hospital, no upper-GI cancer-specific (video)-MDTM was conducted. The duration of an MDTM ranged from 60 to 90 minutes. The MDTM observations were mainly focused on the MDTMs’ structure and organization, conditions such as the ambiance and environment, interaction between the team members, and decision-making processes. Field notes were taken during the MDTM and were summarized at the end of each MDTM (JL). Observations and informal conversations helped build a relationship of trust and were used as inputs for the interviews.

#### Interviews

Semi-structured interviews with clinicians were conducted using a topic-list (**Supplementary Method 1**) based on the expertise of the study core team, and a literature search for studies describing the organization of healthcare protocols and MDTMs (27–29), combined with physician attitudes (30). During all interviews, an opportunity was given to discuss topics that were not part of the topic list, and thus new themes were explored as they evolved during each interview. Broad topics were discussed during the interviews conducted at the first three hospitals. During the study, through iterative analyses of the observations and interviews, the topic-list evolved and encompassed factors contributing to CDM, such as the experienced atmosphere during the MDTM, perceived team dynamics, factors explaining team dynamics, and convictions regarding the feasibility of a certain treatment, which resulted in more focused interviews at the other five hospitals. The mean duration of the interviews was 39 minutes (range, 25–56 minutes). Interviews were audio-recorded and transcribed *ad verbatim* (JL). All interviews were summarized and sent for approval to each clinician to check for correctness.

#### Clinician focus groups

FGs with professionals were conducted in seven of the eight hospitals with three to four clinicians per hospital. The FGs were organized after observations and interviews. Each FG started with a presentation of the most important results of the observations and interviews, followed by a discussion in which the clinicians were invited to explore, complement, or contradict the findings from their institution. FGs were held in a conference room of the hospital (n=3) or *via* videoconference (n=4) due to the SARS-COVID-19 pandemic, and lasted for an average of 90 minutes. The FGs were moderated by JL and observed by PV or RV. In the included referral center, only two clinicians were involved in the clinical pathway of this patient population; thus, the group was too small for a relevant FG. Directly after the FGs, the FG moderator and observer discussed the results of the FG, and thereafter the audio recordings were summarized.

### Data analyses

The data used for the analyses consisted of MDTM field notes, transcripts of the interviews focusing on factors influencing CDM during an MDTM, and summaries of the FGs. A thematic content analysis (23), was used to identify individual and hospital treatment experience that focused on the barriers and facilitators influencing CDM during an MDTM (see **Textbox 1** for a complete overview of the coding process and the identification of emerging themes and subthemes). All themes and subthemes were described in a thematic map for each hospital and an identical thematic map summarizing each theme and subtheme per clinician was made for each individual clinician and each hospital. Analyses of the thematic map resulted in the identification of individual themes and subthemes. Through constant comparison across and within cases (hospitals consisting of teams which participate during an MDTM), associations as well as deviant cases were extracted (24). Furthermore, variability within and between cases were analyzed to explore possible explanations for variabilities in practice. This analysis allowed the identification of two broad types of teams representative of the CDM during an MDTM: guideline-oriented *versus* identification of treatment boundaries (maximizing the perceived likelihood of curation). Both types of hospitals were regarded as two extremes of a continuum. To guarantee anonymity, both types of hospitals are described herein using fictional case descriptions, which are illustrative of each type. Preliminary results were thoroughly discussed by the research team (JL, PV, RV, GN, MW) and thereafter with a research expert in the field of MDTMs (JW).

### Ethics

The Medical Research Ethics Committees of the Netherlands confirmed that ethical approval was not required for this study (W.18.166). The participating hospitals approved this study. Written informed consent was obtained from all the participants prior to the interviews. The participants’ privacy and confidentiality were protected by pseudonymization. The field notes and transcripts were stored pseudonymized for a minimum of 10 years on a secured network of the Netherlands Comprehensive Cancer Organization, and only the core research team members have access to these files.

## Results

### Facilitators and barriers influencing CDM during the MDTM

The different conceptualizations of the clinicians’ perspectives and experience regarding the facilitators and barriers of CDM during an MDTM were described in three themes (**Table 1**):

1) *Team dynamics*, including communication and collaboration during the MDTM, atmosphere during the MDTM, and personal characteristics of the team members, such as their experience and personal treatment objectives;
2) *Patient factors*, including tumor characteristics including the extent of disease and patient characteristics, including physical and cognitive conditions, and the patient’s home situation.
3) *External factors*, including quality indicators, innovation in scientific research, and technical possibilities

**Table 1:** Barriers and facilitators associated with CDM during an oncologic Upper-GI MDTM **Figure 1**.

Theme	Subtheme	Category
Factors influencing team dynamics	Communication and collaboration during the MDTM	Organizational and logistic aspects: (*noon or late afternoon*, *technical efficacy of teleconference, shape and size of MDTM meeting room)* Inter-hospital collaboration *(e.g., training of medical specialist of the referral hospital, providing information regarding patient follow up, MDTM report, video/tele conference versus live attending of MDTM, referral with or without presence of patient’s advocate)*Intra-hospital collaboration *(e.g., low threshold for contacting each other for treatment discussion via telephone or email, informal coffee break, face to face discussion, nonverbal communication, multidisciplinary outpatient clinic visit, prolonged collaboration between clinicians)*
	Atmosphere during MDTM	Communication between clinicians during MDTM: *(e.g., formal or informal, structur*e*d or unstructured, team leader’s style)* Interaction between clinicians during MDTM: *(e.g. respectful, open discussion, constructive, hierarchy, competition, personal characters of attendees, group size, no room for questions, equality)*
	Personal characteristics of team members	Characteristics *(e.g., dominant personalities whom are direct and strong willed versus more timid personalities)* Personal beliefs and experiences *(e.g., learning experience, personal ethics, convictions in the feasibility of a certain treatment, previous experiences with treatment and outcomes)* Personal treatment aims (*e.g., offering hope, ambition to provide more treatment options, being innovative, providing cure, containing and providing quality of life, providing evidence-based medicine)*
Patient factors influencing the treatment proposal	Tumor characteristics	Extent of disease *(e.g., tumor stage, single metastasis, extent and location of positive lymph nodes such as paratracheal or supraclavicular lymph nodes, cT4b tumor stage, most variation in cases of doubt)*
	Patient characteristics	Physical and cognitive condition *(e.g., severity of comorbidities, prognosis, calendar age versus physical condition in relation to calendar age, intellectual ability, paraneoplastic weight loss)* Sociodemographic *(e.g., patient’s family, safety net, domestics)* Patient’s intrinsic motivation *(e.g., motivation towards the proposed treatment plan, mindset in life, patient’s preference known versus unknown during MDTM)*
External factors influencing the treatment proposal	Quality indicators	Possible influence of auditing regarding treatment outcomes *(e.g., refraining from surgery in patients with multimorbidity since it might negatively influence the proportion of postoperative complications)* Possible reaching targets for volume standards for surgery *(e.g., offering surgery in patients with multimorbidity in order to achieve the minimal number of required case, during period when centralization just started)*
	Innovation in scientific research	Team’s ambition for innovation *(e.g., experimental treatment options, testing the limits of treatment within/outside scientific research)* Collaboration in scientific research *(e.g., international collaboration, national collaboration, well informed about developments and ongoing studies)*
	Innovation in technical possibilities in surgery	Laparoscopic minimal invasive surgery *(e.g., offering minimal invasive resection in multimorbid patients)* Robot assisted surgery *(e.g., removing more extended lymph nodes)*

Variability in the organization of an expert MDTM.

### Team dynamics

#### Structure and organization

**Figure 1:**
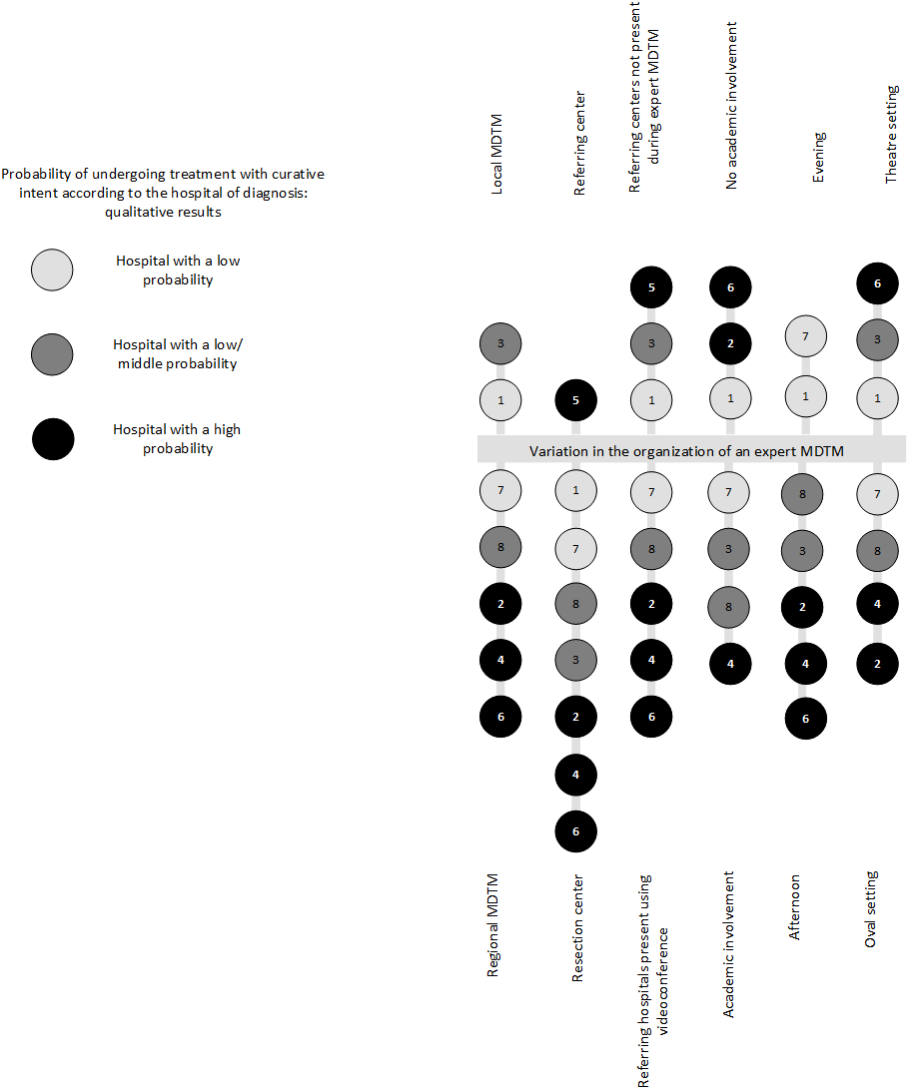
Variability in the organization of an expert MDTM. All participating hospitals are displayed in this figure. Each included hospital is represented in the form of a circle, including a hospital number. In addition, the probability groups are represented by different shades of gray. The referring hospital is only partly represented in this figure. MDTM, multidisciplinary team meeting.

Resection hospitals organized their MDTMs at noon (n=5) or in the late afternoon (n=2) (**Figure 1**). In most hospitals (n=4), the MDTM room layout was an oval- or U-shaped setting, in which the whole team was facing each other and was within adequate sight of the TV monitor, as opposed to the theater set-up at the two other hospitals. One clinician stated that an oval- or U-shaped setting benefited communication and discussion during the MDTM.

Observations of MDTMs, showed that in most hospitals all referring hospitals participated in the meetings by videoconference over the entire MDTM session, whereas at others, the referring hospitals did not participate in the MDTM (n=2), while others alternated during the videoconference and were only present when their own patients were discussed (n=1). During one MDTM, the referring hospitals were physically present during the MDTM. Some clinicians mentioned that collaboration between centers improved by in-person attendance at the MDTM and facilitated team discussion. Non-verbal communication and face-to-face discussions were considered valuable.

The timing and visibility of writing the minutes of the MDTM advice differed between hospitals. In three MDTMs, the clinical advice was documented live and was visible during the MDTM and all present clinicians could complement, adjust, and agree with the advice. In most other MDTMs, the advice was documented by a secretary during the MDTM and verified by the chairman after the MDTM. Documenting the minutes of the MDTM advice live during the MDTM was indicated to facilitate multidisciplinary adjustment and was considered beneficial by some clinicians.

#### Collaboration and communication during the MDTM

In the MDTMs in which referring clinicians participated, most clinicians mentioned that collaboration between the resection and referral hospitals (inter-hospital communication) improved over time, as they became better acquainted due to collaboration during the expert MDTM, as explained by a medical oncologist: *“The advantage of the collaboration during a MDTM is that you meet each medical oncologist in the region, and communication is very easy” (*Medical oncologist-5, High).

The clinician that met the patient during the outpatient clinic visit may not always participate during the MDTM, meaning that at times the referring hospital presenting the case, has never met the patient.

An excessively large group size was indicated as a barrier for a successful CDM, since it was more difficult to follow and comprehend the treatment discussion. Technical aspects of videoconference during the MDTM were reported to influence team discussion and were especially considered as barriers when equipment was inadequate.

#### Atmosphere during the MDTM

The atmosphere during MDTM, such as the culture and setting, differed across institutions. In MDTMs with an informal atmosphere, the meeting environment was relaxed, and discussion tended to be unstructured, possibly due to a team leader who did not fulfill a leadership role. In other MDTMs, the atmosphere was more formal, with a team leader who facilitated and enhanced discussion during the MDTM. To highlight the influence of the meeting atmosphere during the MDTM, these details were added to the case descriptions (**Textbox 2**, **Textbox 3**). When clinicians were not on time for the MDTM, in the event of repeated interruptions from phone calls, or when the technical quality of the teleconference was low, the structure and progress of the MDTM became impaired.

Textbox 2 **Case illustration of a guideline-oriented hospital.**

Example of a hospital with a low probability of proposing treatment with curative intent, based on observations and interviews across multiple hospitals at a similar end of the continuum (guideline-oriented, quality of life-oriented *[e.g., less invasive treatment approach])*

The MDTM takes place once a week in the late afternoon. During the MDTM all types of gastrointestinal cancer patients are discussed. Teleconferencing is used to discuss patients from referring hospitals. During the MDTM a clear structured discussion is lacking, and the treatment plan and conclusion are not summarized at the end of each discussion. The overall impression of the MDTM’s atmosphere is informal. The team is oriented towards an optimal balance between following the guidelines and proposes treatment with curative intent, whilst taking the patient’s quality of life into consideration. Eligibility for patient participation in scientific research is mentioned in the minority of the discussed patients during the MDTM.

The team uses guidelines for treatment decision-making: *e.g., “I have the feeling that most of the time the guideline is followed” MO1, L*. The team members feel that the treatment decision should be made based on evidence: *e.g.*, “*Well I think there are hospitals who believe surgery is the holy grail, and there are hospitals who think that removing all lymph nodes is the best option…there is absolutely no evidence for that.” S8, L.* For each case, expected quality of life is taken highly into consideration and a guideline-based personalized treatment plan is proposed: *e.g., “I think that we take the interest of the patient into account, or the patient’s opinion, what does the patient want, and I can imagine that there are hospitals that are more guideline-based” S8, L.* The team’s convictions that in borderline cases the extent of the disease is only the tip of the iceberg and thus since prognosis will probably be limited, treatment should be achievable and quality of life during the treatment modality should be prioritized: *e.g., “When estimating the treatment decision in doubtful cases, surgery or definitive chemoradiation, or perhaps a bit less chemo, or maybe just palliation, or refraining from treatment? Quality of life plays a major role” RO3, M.*

Patient’s frailty, comorbidities, and calendar age are taken into account during treatment decision-making and their impact is carefully weighed against the expected effect on quality of life. The team is cautious in proposing invasive treatment and weighs the treatment effects on the expected prognosis and impact on the patient’s quality of life. In elderly patients diagnosed with multimorbidity, often primary resection in gastric cancer, definitive chemoradiation in esophageal cancer or palliative treatment is considered: *e.g. “Neoadjuvant chemotherapy in gastric cancer is a treatment with high impact on quality of life and in elderly, the threshold is lower and less is required” MO7, L.* In a young healthy patient diagnosed with a T4b tumor or a solitary metastasis, in most cases the team proposes palliative therapy: *e.g., “they hold a conservative approach since in the majority of the cases, it is the tip of the iceberg” CM7, L*. Thus, the team limits their search for treatment boundaries: *e.g., “During discussion there is no right or wrong decision, since apparently there is no clear evidence, which means that it is more of an expert opinion, and therefore, the discussion relies on good arguments, since there is no back-up from literature” S8, L*. Although the team is quality of life-oriented and proposes less invasive treatment options, they follow the guidelines and propose treatment with curative intent in potentially curable patients.

Case description consists of a combination of multiple cases with a similar typology in order to guarantee anonymity.

Case manager (CM), surgeon (S), gastroenterologist (GE), medical oncologist (MO), radiation oncologist (RO).

Hospital with a low probability of proposing treatment with curative intent (L), Hospital with a middle probability of proposing treatment with curative intent (M). For example: MO1,L stands for medical oncologist, interviewed in hospital 1, which is a hospital with a low probability of proposing treatment with curative intent.

Textbox 3 **Case illustration of hospital search for treatment boundaries.**

Example of a hospital with a high probability of proposing treatment with curative intent, based on observations and interviews in multiple hospitals at a similar end of the continuum (searching for treatment boundaries aiming for a cure, more invasive treatment approach).

The MDTM takes place once a week during the lunch break. During the MDTM only esophagogastric cancer patients are discussed. Teleconferencing is used to discuss patients from referring hospitals. During the MDTM the discussion is structured and the conclusion and treatment plan are summarized after the discussion by the team leader. The overall impression of the MDTM’s atmosphere is formal. The team is oriented towards an optimal balance between proposing treatment with curative intent, searching for the boundaries of treatment, and the use of experimental treatment regimens whilst taking the patient’s personal situation in consideration.

The team feels that guidelines are merely a guide and thus they feel that they can be innovative and more often aim for treatment with curative intent: *e.g., “You need a certain drive, a certain motivation, to make those steps, and it cannot be expected that everyone takes that risk… I believe that you have to take steps to improve the survival of this group”. S4, H.* The teams feels that participation in scientific research is of importance: *e.g., “I believe that we ought to make steps in order to improve the inferior survival for this patient group, and therefore for instance I believe in the HIPEC treatment. More research needs to be conducted for patients with gastric cancer” S4, H*. Additionally, with increasing expertise, the team’s ambition increases in searching for treatment options maximizing chances of curation: *e.g., “You get the feeling, that if you have seen a lot of cases you can and may search for boundaries and deviate from the guideline” MO2, H*. Clinicians feel that providing hope has a positive effect on quality of life and prognosis: *e.g., “If you can give someone a glimmer of hope, it makes them live longer and improves their quality of life” S2, H.*

Patient’s frailty, comorbidities, and age are taken into account during treatment decision-making and their impact is carefully weighed against the possibility of undergoing treatment with curative intent: *e.g., “I think that the variance exists mostly in the potentially metastasized, comorbid, aged, and if you know more about that, if you have more data about it, you can make better decisions” MO6, H*. The team is cautious in prematurely refraining from treatment with curative intent and weighs the feasibility of more invasive treatment options on the expected prognosis and impact on the patient’s quality of life. In elderly patients diagnosed with multiple morbidities other disciplines are consulted in order to identify if invasive treatment might still be feasible (e.g., anesthesiologist, geriatrician): *e.g., “So we are always searching for possible treatment combinations, multidisciplinary, to provide patients with a curative treatment option, and I can imagine that not all hospitals do that. They might not all have specialists in their center, and not the expertise, then automatically you are inclined to refrain from surgery” S4, H*. In young patients diagnosed with a T4b tumor, the team considers downstaging of the tumor, potentially facilitating a curative treatment option: *e.g., “We are always searching for an opportunity of cure, which means, T4B patients with growth into adjacent organs, first chemoradiotherapy, downstaging, or patients with oligometastatic disease, solitary metastasis, whether the biological behavior of the tumor might make surgical resection possible” S4, H .* The team holds similar opinions regarding solitary metastasis: *e.g., “A fit patient presenting with oligometastatic disease, I would not feel restrained and would discuss a resection with the patient and I think that others are more conservative and believe that it is metastatic disease and propose palliative treatment” S6, H.* Although the team opts for more invasive treatment approaches they do consider the patient’s quality of life.

Case description consist of a combination of multiple cases in order to guarantee anonymity

Gastroenterologist (GE), Radiation oncologist (RO), Surgeon (S), Medical oncologist (MO)

Hospital with a high probability of proposing treatment with curative intent (H), Hospital with a middle probability of proposing treatment with curative intent (M)

Most clinicians reported that most of the time during the MDTM, team discussion elapsed respectfully, was open and egalitarian, and the MDTM was considered as a safe environment in which critical thinking was promoted. However, some clinicians felt that there was not always room for questions, especially if the workload due to the number of cases was high during the MDTM, as explained by a radiation oncologist: *“The atmosphere differs, sometimes it can be hasty, and when you ask a difficult question, or if you disagree, you have to kick your own butt to ask that difficult question even though they start to groan” (*Radiation oncologist-6, High).

Furthermore, some clinicians stated that opinions of senior clinicians could be decisive during treatment discussion, and not everyone’s opinion was regarded as equally important: *“Everyone has a role, [ … ] some have more authority than others”* [Surgeon-3, Middle].

#### Personal factors of team members

One of the interviewees explained that the combination of the attending clinicians played a decisive role during CDM: *“It* (decision making) *depends on the combination of doctors during an MDTM, but that happens everywhere. Some search for treatment boundaries, looking for the benefits of doubt. At times, I feel that if that specific patient was discussed in another MDTM that patients might have received a different treatment proposal* (more invasive)*.”* [Gastroenterologist-1, low] The personal characteristics of clinicians were pivotal during CDM as described by another interviewee: *“There are MDTMs in which certain people dominate the whole meeting and their dominance can be more decisive than the individual opinions of each medical specialist. [ … ] However, if during the MDTM discussion, they hold themselves back and don’t stand up against the more dominant personalities, room for dominance during an MDTM is created”* [Gastroenterologist-3, Middle]. This was also mentioned by a case manager: *“I think that everyone can give their opinion, but it is very individual, medical oncologists especially, their input depends on the present specialist” (*Case manager-1, Low).

Furthermore, the physician’s personal aims and convictions in the feasibility of a certain treatment for a particular patient played an important role in CDM process: *“Believing in a certain treatment [ … ] you have done well, you have prolonged their life span and increased their quality of life (QoL), but there are also pessimists in life, who think: “why would you do everything”“* [Gastroenterologist-8, Middle]. Being ambitious and innovative in searching for treatment boundaries were described as facilitators for more invasive treatment suggestions, as described by a surgeon: *“You need a certain drive, a certain motivation, to make those steps, and it cannot be expected that everyone takes that risk [ … ] I believe that you have to take steps to improve the survival for this group”.* [Surgeon-4, High] However, other clinicians aimed to be more conservative and regarded QoL of the patient more important: *“We feel that with the more conservative approach, patients are protected for too aggressive treatments, and I completely endorse that.”* [Medical Oncologist-1, Low] .

Previous experience played a role in CDM, and the impact of this experience was stated to be dependent on the clinician’s convictions. One clinician explained that in the past few years their treatment outcomes have not always been optimal: *“In our hospital gastroenterological oncology has not always been top-notch, and undoubtfully that will have played a role in decision-making”* [Gastroenterologist-8, Middle]. In addition, the occurrence of recent experience with postoperative morbidity was mentioned as a factor, which might have led to a more cautious approach in further CDMs as explained by a surgeon: *“If during a specific period, a patient’s outcome was worse than before, it does not necessarily need to be anastomotic leakage, it can also be something different, with perioperative problems, you become more critical about surgery”* [Surgeon-2, High].

### An illustration of a continuum – guideline-oriented versus searching for boundaries

Ultimately, all clinicians would propose curative treatment for patients with a potentially curable disease stage. Differences in practice occurred most often in borderline cases (e.g., elderly fragile patients, solitary metastasis, or cT4b tumor stage). In principle, all hospitals followed guidelines, used evidence-based medicine, and considered patient characteristics such as age, comorbidity, and QoL. Nevertheless, hospitals differed in the extent and degree in which these factors were taken into account, as described in **Textbox 2**, **3**.

Based on these observations, roughly two ends of the continuum could be described based on the differences in proposing treatment with curative intent. To guarantee anonymity, the case descriptions in **Textbox 2**, **3** are fictitious cases, serving as illustrations of both ends of the continuum.

At one end of the continuum, clinicians are more inclined to strictly follow treatment guidelines. Clinicians felt that the effect of the treatment on the QoL was an important factor in the CDM, and that the patient should be protected from any unnecessary morbidity; therefore, a more conservative approach was proposed (see **Textbox 2** for case description). However, on the other end of the continuum, the clinicians were more inclined to propose more invasive and innovative treatment approaches in borderline cases. They believed that offering treatment with curative intent gave the patient hope, and ultimately contributed to a better QoL (see **Textbox 3** for the case description).

## Discussion

This qualitative multiple case study aimed to identify the facilitators and barriers occurring during the CDM during a tumor-specific upper-GI cancer MDTM and illustrates factors explaining differences in local hospital practice. CDM during an MDTM is influenced by team dynamics, such as meeting atmosphere, personal characteristics of participants, communication between team members, patient characteristics, and external factors, such as quality indicators, innovation in scientific research and innovation in technical possibilities. Teams differed in the extent and methods in which they took the guidelines into account. At one end of the continuum, teams tended towards a more guideline-oriented approach and focused more on preserving a patient’s quality of life, versus the opposite end, teams tended towards decisions maximizing cure and proposing invasive treatment more often. Along this continuum, QoL was considered during the decision-making process, although opinions regarding QoL varied from preserving (more conservative approach) to providing QoL (more invasive approach).

The present study demonstrates that CDM is influenced by the quality of team dynamics during an MDTM, which increases due to good communication, conclusive discussion, adequate leadership, and a climate of respect between team members. In addition, it was observed that in balanced teams with a climate of respect between team members, clinicians were mutually critical regarding each other’s treatment suggestions, thereby facilitating successful CDM. Nevertheless, in some teams, clinicians felt that offering one’s view during the discussion was not always appreciated, which was experienced as a barrier to adequate CDM. Adequate leadership of the MDTM and participation of all relevant team members during the discussion has been reported by previous studies to influence team dynamics (17–19). Important links between teamwork and performance in error avoidance have been demonstrated by aviation studies (31). The root cause of aviation accidents was attributed to failures of: leadership, decision-making, and communication (32). Moreover, team dynamics and trust among clinicians has been identified in multiple studies as a fundamental principle of error reduction (33, 34).

Improvement in team dynamics including leadership, a constructive culture of debate and a psychologically safe atmosphere during the MDTM could be achieved by implementing the process of crew resource management (CRM). CRM facilitates situational awareness leading to enhanced learning, promotes desired behavioral changes, and produces positive reactions (35, 36). CRM has been shown to be effective in aviation (32), and in medicine at the emergency department (37) and in the operating theater (38, 39), and thus might hold the potential to improve team dynamics, performance, and leadership during an MDTM. Furthermore, future research could assess the extent to which CRM improves clinical decision making.

The results of the current study demonstrate that there are teams that are more inclined to offer a more invasive treatment approach (e.g., offering treatment with curative intent), whereas other teams hold a more conservative attitude (e.g., adhering to guidelines). As described in 1975 by Haney, physicians have been characterized as tending towards health maintenance, including QoL, or as tending towards interventions (e.g., invasive treatment) (40). The clinician tending towards QoL was more likely to be adherent to guidelines preserving QoL, whereas the interventionist seems more likely to be disease oriented and is inclined toward immediate action improving QoL (40). In concordance with previous research (8, 41–43), the results of the current study illustrate that these different approaches might be attributed to differences in clinician’s characteristics, previous treatment experiences, and their convictions regarding the feasibility of a certain treatment for a specific patient. Therefore, depending on the clinician’s personality and convictions regarding a certain treatment and its outcomes, differences between guidelines and practice may occur when clinicians, and thus teams hold different views, and develop a different pattern of knowledge based on their previous experience (14).

Furthermore, our findings showed that clinicians seemed to have different perceptions regarding preserving and improving QoL. Some clinicians felt that QoL was provided by giving hope for survival by offering treatment possibilities that would increase the chance of survival, whereas others felt that QoL needed to be preserved by offering less invasive treatment regimens. This might potentially shed light on the variability of hospital practices regarding the probability of proposing treatment with curative intent. Hence, during the MDTM and outpatient clinic visits the patient’s QoL perceptions should be taken into account.

Clinical guidelines facilitate the delivery of evidence-based high-quality care (44, 45). Obviously, guidelines should leave room for personalized medicine, yet our study demonstrated that differences in hospital practice varied in terms of the application of guidelines, especially in borderline cases (i.e., older patients, patients with solitary metastasis, and patients with cT4b tumor stage). Especially for the borderline cases, a golden standard might be lacking and may not be described in the current guidelines yet. Hence, this variability could be attributed to the described continuum, since clinicians dealt differently with borderline cases based on their personality and previous experience, resulting in more conservative or more invasive treatment proposals. Nevertheless, the MDTM provides a treatment advise, the treatment decision is made during the outpatient clinic visit using shared decision making. the Furthermore, future research could assess the extent to which practice variation between hospitals is observed in borderline cases, which can be achieved by presenting real-life clinical cases to multidisciplinary teams. Additionally, further research may explore case-related information, and elucidate common day practice in this patient group.

The main strength of this study is the combination of observations and interviews, which provides a broad and in-depth understanding of the factors influencing CDM during MDTMs. Additionally, this study consists of observations of multiple expert tumor specific MDTMs reflecting deviant case sampling (20). Furthermore, the reliability and validity of the data increased due to data triangulation (i.e., using multiple data sources to develop a comprehensive understanding) and the summaries serving as member checks (46, 47). Nevertheless, there are some limitations to consider while interpreting the results. Since all observations and interviews were carried out by a single researcher, researcher bias might have occurred. However, peer debriefing was conducted during the period of data collection and analyses, facilitating reflection, which can be considered a strength of this study. Another limitation might be that some of the FGs were conducted *via* videoconference, which can be hypothesized that this might have impaired FG discussion.

In conclusion, this study investigated the factors influencing variations in team dynamics found to influence CDM during an MDTM. Potentially attributing to variability in practice. Adequate leadership, conclusive discussion and a climate of respect between team members during an MDTM is essential for CDM. Some teams tended to search for decisions maximizing chances of cure and proposed invasive treatment more often, whereas other hospitals tended to be more guideline-oriented and focused more on preserving a patient’s QoL. This study contributes to the awareness that variation in team dynamics influences CDM during an MDTM.

## Data availability statement

The raw data supporting the conclusions of this article will be made available by the authors, without undue reservation.

## Ethics statement

The studies involving human participants were reviewed and approved by the Medical Research Ethics Committees of the Netherlands (W.18.166). The patients/participants provided their written informed consent to participate in this study.

## Author contributions

All authors listed have made a substantial, direct, and intellectual contribution to the work and approved it for publication.

## Funding

This study was funded by a grant from the Dutch Cancer Society (Project No. 10895). The funders had no role in the study design, data collection and analysis, preparation of the manuscript or decision to publish.

## Acknowledgments

The authors thank the registration team of the Netherlands Comprehensive Cancer Organization (IKNL) for the collection of data for the NCR, as well as the participating clinicians for their time and openness during the interviews and focus groups. The authors thank Wiley Editing Services for editing of the manuscript (e.g., copy editing, grammatical assistance, editorial assistance). Wiley Editing Services were financially compensated for their editing. This study was not registered.

## Conflict of interest

Author PS received research support or funding from EndoStim, Pentax, Norgine, Motus GI and The Enose company and is a member of the Advisory Board of Motus GIE. Author HL is a consultant or adviser in BMS, Lilly, MSD, Nordic Pharma, Servier, and received research funding and/or medication supply from Bayer, BMS, Celgene, Janssen, Lilly, Nordic Pharma, Philips, Roche, Servier. Author RV: received research grants from Roche and Bristol-Myers Squibb.

The remaining authors declare that the research was conducted in the absence of any commercial or financial relationships that could be construed as a potential conflict of interest.

## Publisher’s note

All claims expressed in this article are solely those of the authors and do not necessarily represent those of their affiliated organizations, or those of the publisher, the editors and the reviewers. Any product that may be evaluated in this article, or claim that may be made by its manufacturer, is not guaranteed or endorsed by the publisher.

## References

[1] Bray F Ferlay J Soerjomataram I Siegel RL Torre LA Jemal A. Global cancer statistics 2018: GLOBOCAN estimates of incidence and mortality worldwide for 36 cancers in 185 countries. CA Cancer J Clin (2018) 68:394–424. doi: 10.3322/caac.21492. 30207593

[2] Lordick F Mariette C Haustermans K Obermannova R Arnold D. Oesophageal cancer: Esmo clinical practice guidelines for diagnosis, treatment and follow-up. Ann Oncol (2016) 27:v50–7. doi: 10.1093/annonc/mdw329 27664261

[3] Smyth EC Verheij M Allum W Cunningham D Cervantes A Arnold D. Gastric cancer: Esmo clinical practice guidelines for diagnosis, treatment and follow-up. Ann Oncol (2016) 27:v38–49. doi: 10.1093/annonc/mdw350 27664260

[4] van Putten M Nelen SD Lemmens VEPP Stoot JHMB Hartgrink HH Gisbertz SS. Overall survival before and after centralization of gastric cancer surgery in the netherlands. Br J Surg (2018) 105:1807–15. doi: 10.1002/bjs.10931 30132789

[5] Luijten J Vissers PAJ Lingsma H Leeuwen N Rozema T Siersema P. Changes in hospital variation in the probability of receiving treatment with curative intent for esophageal and gastric cancer. Cancer Epidemiol (2021) 71:101897. doi: 10.1016/j.canep.2021.101897 33484974

[6] van Putten M Koeter M van Laarhoven HWM Lemmens VEPP Siersema PD Hulshof MCCM. Hospital of diagnosis influences the probability of receiving curative treatment for esophageal cancer. Ann Surg (2018) 267:303–10. doi: 10.1097/SLA.0000000000002063 27811508

[7] van Putten M Verhoeven RH van Sandick JW Plukker JT Lemmens VE Wijnhoven BP. Hospital of diagnosis and probability of having surgical treatment for resectable gastric cancer. Br J Surg (2016) 103:233–41. doi: 10.1002/bjs.10054. 26621158

[8] Wennberg JE. Dealing with medical practice variations: A proposal for action. Health Aff (Millwood) (1984) 3:6–32. doi: 10.1377/hlthaff.3.2.6. 6432667

[9] Gravesteijn BY Sewalt CA Ercole A Lecky F Menon D Steyerberg EW. Variation in the practice of tracheal intubation in europe after traumatic brain injury: A prospective cohort study. Anaesthesia (2020) 75:45–53. doi: 10.1111/anae.14838. PMC7344983 31520421

[10] van Hagen P Spaander MC van der Gaast A Van Rij CM Tilanus HW Van Lanschot JJ. Impact of a multidisciplinary tumour board meeting for upper-gi malignancies on clinical decision making: A prospective cohort study. Int J Clin Oncol (2013) 18:214–9. doi: 10.1007/s10147-011-0362-8 22193638

[11] Trip AK Stiekema J Visser O Dikken JL Cats A Boot H. Recent trends and predictors of multimodality treatment for oesophageal, oesophagogastric junction, and gastric cancer: A dutch cohort-study. Acta Oncol (2015) 54:1754–62. doi: 10.3109/0284186X.2015.1009638 25797568

[12] Lamb BW Brown KF Nagpal K Vincent C Green JS Sevdalis N. Quality of care management decisions by multidisciplinary cancer teams: A systematic review. Ann Surg Oncol (2011) 18:2116–25. doi: 10.1245/s10434-011-1675-6. 21442345

[13] Selby P Popescu R Lawler M Butcher H Costa A. The Value and Future Developments of Multidisciplinary Team Cancer Care. Am Soc Clin Oncol Educ Book (2019) 39:332–40. doi: 10.1200/EDBK_236857 31099640

[14] Bate L Hutchinson A Underhill J Maskrey N. How clinical decisions are made. Br J Clin Pharmacol (2012) 74:614–20. doi: 10.1111/j.1365-2125.2012.04366.x PMC3477329 22738381

[15] Soukup T Petrides KV Lamb BW Sarkar S Arora S Shah S. The anatomy of clinical decision-making in multidisciplinary cancer meetings: A cross-sectional observational study of teams in a natural context. Med (Baltimore) (2016) 95:e3885. doi: 10.1097/MD.0000000000003885. PMC4998467 27310981

[16] National Cancer Action Team. Characteristics of an effective MDT. (2010) London: National Cancer Action Team.

[17] Taylor C Atkins L Richardson A Tarrant R Ramirez AJ. Measuring the quality of mdt working: An observational approach. BMC Cancer (2012) 12:202. doi: 10.1186/1471-2407-12-202 22642614 PMC3489862

[18] Haward R Amir Z Borrill C Dawson J Scully J West M Sainsbury R. Breast cancer teams: The impact of constitution, new cancer workload, and methods of operation on their effectiveness. Br J Cancer (2003) 89:15–22. doi: 10.1038/sj.bjc.6601073. PMC2394209 12838294

[19] Jalil R Akhter W Lamb BW Taylor C Harris J Green JS Sevdalis N. Validation of team performance assessment of multidisciplinary tumor boards. J Urol (2014) 192:891–8. doi: 10.1016/j.juro.2014.03.002 24631109

[20] Patton MQ. Qualitative evaluation and research methods. Thousasnd Oaks, CA: Sage (1990).

[21] Strauss AL Corbin JM. Grounded Theory Research: Procedures, Canons, and Evaluative Criteria. Thousand Oaks: Sage Publications (1990).

[22] Korstjens I Moser A. Series: Practical guidance to qualitative research. part 4: Trustworthiness and publishing. Eur J Gen Pract (2018) 24:120–4. doi: 10.1080/13814788.2017.1375092 PMC8816392 29202616

[23] Green J TN. Analysing qualitative data. In: Silverman D, editor. Qualitative methods for health research, 1st edn. London: Sage Publications (2004). p. 173–200.

[24] Yin RK. Case study research: Design and methods. Thousand Oaks, CA: Sage (1994).

[25] Green T. Qualitative methods for health research. (2014), 121–122. chapter 4.

[26] Patton MQ. Qualitative research and evaluation methods. Thousand Oaks, CA: 3rd Sage Publications (2002).

[27] Davies AR Deans DA Penman I. The multidisciplinary team meeting improves staging accuracy and treatment selection for gastro-esophageal cancer. Dis Esophagus (2006) 19:496–503. doi: 10.1111/j.1442-2050.2006.00629.x 17069595

[28] Du CZ Li J Cai Y Sun YS Xue WC Gu J.. Effect of multidisciplinary team treatment on outcomes of patients with gastrointestinal malignancy. World J Gastroenterol (2011) 17:2013–8. doi: 10.3748/wjg.v17.i15.2013 PMC3082756 21528081

[29] Siemerink EJ Schaapveld M Plukker JT Mulder NH Hospers GA. Effect of hospital characteristics on outcome of patients with gastric cancer: A population based study in north-east netherlands. Eur J Surg oncology: J Eur Soc Surg Oncol Br Assoc Surg Oncol (2010) 36:449–55. doi: 10.1016/j.ejso.2010.03.011 20399068

[30] Thornblade LW Truitt AR Davidson GH Flum DR Lavallee DC. Surgeon attitudes and practice patterns in managing small bowel obstruction: A qualitative analysis. J Surg Res (2017) 219:347–53. doi: 10.1016/j.jss.2017.06.052 PMC5679108 29078904

[31] Helmreich R Giutahw SJ Childress T eds. IDR. Aldershot, UK: Ashgate Publishing Limited (2004).

[32] Helmreich RL Merritt AC Wilhelm JA. The evolution of crew resource management training in commercial aviation. Int J Aviat Psychol (1999) 9:19–32. doi: 10.1207/s15327108ijap0901_2 11541445

[33] Leape LL KA Berwick DM. Reducing adverse drug events and medical errors. Boston: Institute for Healthcare Improvement (1998).

[34] Kohn LTCJ Donaldson MS eds. To err is human. In: Building a safer health care system. Washington DC: National Academy Press. 25077248

[35] Salas E Burke CS Bowers CA Wilson KA. Team training in the skies: Does crew resource management (crm) training work? Hum Factors (2001) 43:641–74. doi: 10.1518/001872001775870386 12002012

[36] Salas E Rhodenizer L Bowers CA. The design and delivery of crew resource management training: Exploiting available resources. Hum Factors (2000) 42:490–511. doi: 10.1518/001872000779698196 11132810

[37] McConaughey E. Crew resource management in healthcare: The evolution of teamwork training and medteams. J Perinat Neonatal Nurs (2008) 22:96–104. doi: 10.1097/01.JPN.0000319095.59673.6c 18496068

[38] Wakeman D Langham MR Jr. Creating a safer operating room: Groups, team dynamics and crew resource management principles. Semin Pediatr Surg (2018) 27:107–13. doi: 10.1053/j.sempedsurg.2018.02.008 29548351

[39] Savage C Gaffney FA Hussain-Alkhateeb L Olsson Ackheim P Henricson G Antoniadou I. Safer paediatric surgical teams: A 5-year evaluation of crew resource management implementation and outcomes. Int J Qual Health Care (2017) 29:853–60. doi: 10.1093/intqhc/mzx113. 29024977

[40] CA H. Psychosocial factors involved in medical decision making. In: Millon T, editor. Medical behavioral sciences. WB Saunders: Philadelphia (1975). p. 420–32.

[41] Neuman GAWS Christiansen ND. The relationship between work-team personality composition and the job performance of teams. Group Organ Manage (1999), (24) 28–45. doi: 10.1177/1059601199241003

[42] Barry B Stewart GL. Composition, process, and performance in self-managed groups: The role of personality. J Appl Psychol (1997) 82:62–78. doi: 10.1037/0021-9010.82.1.62 9119798

[43] Hajjaj FM Salek MS Basra MK Finlay AY. Non-clinical influences on clinical decision-making: A major challenge to evidence-based practice. J R Soc Med (2010) 103:178–87. doi: 10.1258/jrsm.2010.100104 PMC2862069 20436026

[44] Hunter B Segrott J. Re-mapping client journeys and professional identities: A review of the literature on clinical pathways. Int J Nurs Stud (2008) 45:608–25. doi: 10.1016/j.ijnurstu.2007.04.001 17524406

[45] Panella M Marchisio S Di Stanislao F. Reducing clinical variations with clinical pathways: Do pathways work? Int J Qual Health Care (2003) 15:509–21. doi: 10.1093/intqhc/mzg057. 14660534

[46] Britten N. Qualitative interviews in medical research. BMJ (1995) 311:251–3. doi: 10.1136/bmj.311.6999.251. PMC2550292 7627048

[47] Sim J Sharp K. A critical appraisal of the role of triangulation in nursing research. Int J Nurs Stud (1998) 35:23–31. doi: 10.1016/S0020-7489(98)00014-5 9695007

