# Supplementary material for: Team dynamics and clinician’s experience influence decision-making during Upper-GI multidisciplinary team meetings: A multiple case study
Source: Front Oncol. 2022 Oct 18;12:1003506. doi: 10.3389/fonc.2022.1003506 (PMC9623154; doi:10.3389/fonc.2022.1003506)
Supplement: Supplementary file 1 [file DataSheet_1.docx]

Supplementary Methods 1: Interview guide

Introduction of interview

Initial topic list

Organizational context

Considerations in care provided (patients notes as a reminder):

• Organization of clinical pathway

• Patient discussion (MDTM and with colleagues)

• Are all patients discussed in a MDTM

Collaboration in region

• Logistics

• Financial aspects

• Political problems

• Referral

• Processes

• Collaboration between centers

Treatment decision-making:

• Including: reception, processes, and influences on treatment

Knowledge

• Physician/hospital

• Conferences

• Centralization and knowledge drainage

Physician’s preferences

• Recent positive or negative experience with sort of treatment

• Observed referral difference between colleagues

Patient related factors

• Treatment options

• Shared decision-making

• Cases of doubt

• Referral

Considerations in treatment decisions
